# *catena*-Poly[[[aqua(7-hydroxy-2*H*-1-benzopyran-2-one)sodium]-di-μ-aqua] 2-oxo-2*H*-1-benzopyran-7-olate monohydrate]

**DOI:** 10.1107/S160053681002341X

**Published:** 2010-06-23

**Authors:** Fuchun Zheng, Yicun Chen, Jia Ni, Jinzhi Wang, Ganggang Shi

**Affiliations:** a Department of Pharmacy, First Affiliated Hospital, Shantou University Medical College, Shantou 515041, People’s Republic of China; b Department of Pharmacology, Shantou University Medical College, Shantou 515041, People’s Republic of China; c Analysis and Testing Center, Shantou University, Shantou 515041, People’s Republic of China; d Department of Chemistry, Shantou University Medical College, Shantou 515041, People’s Republic of China

## Abstract

The asymmetric unit of the title compound, {[Na(C_9_H_6_O_3_)(H_2_O)_3_](C_9_H_5_O_3_)·H_2_O}_*n*_, contains two crystallographically independent Na atoms, two 7-hy­droxy­coumarin ligands, six coordinated water mol­ecules, two 7-hy­droxy­coumarin anions and two uncoordinated water mol­ecules. Both Na atoms exhibit a distorted octa­hedral coordination geometry and are coordinated by five water O atoms and the terminal O atom from a 7-hy­droxy­coumarin ligand. Four of the water mol­ecules are bridging, whereas the fifth is terminal. Na—O bond distances are in the range 2.288 (2)–2.539 (2) Å. In the chains, extending parallel to [100], adjacent Na atoms are separated by 3.60613 (7) Å. The uncoordinated water mol­ecules and 7-hy­droxy­coumarin phenolate anions are located between the chains and are hydrogen bonded to the chains.

## Related literature

For applications of the active drug umbelliferone (7-hy­droxy­coumarin, 7-*HOC*), see: Toyama *et al.* (2009 ▶); Egan *et al.* (1990 ▶). For applications of metal complexes of coumarin, see: Nath *et al.* (2005 ▶).
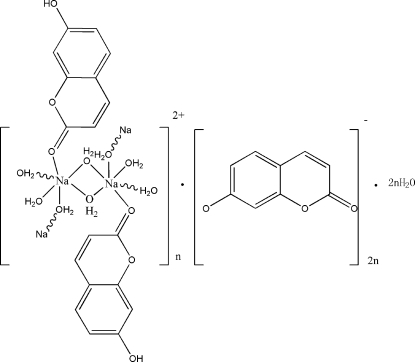


## Experimental

### 

#### Crystal data

[Na(C_9_H_6_O_3_)(H_2_O)_3_](C_9_H_5_O_3_)·H_2_O
*M* *_r_* = 836.64
Monoclinic, 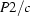

*a* = 11.7803 (5) Å
*b* = 7.6462 (3) Å
*c* = 42.3249 (17) Å
β = 102.184 (1)°
*V* = 3726.5 (3) Å^3^
*Z* = 4
Mo *K*α radiation
μ = 0.14 mm^−1^
*T* = 173 K
0.46 × 0.44 × 0.39 mm

#### Data collection

Bruker SMART 1000 CCD diffractometer
Absorption correction: multi-scan (*SADABS*; Sheldrick, 2004 ▶) *T*_min_ = 0.938, *T*_max_ = 0.947
16682 measured reflections
7251 independent reflections
5790 reflections with *I* > 2σ(*I*)
*R*_int_ = 0.027

#### Refinement

*R*[*F* ^2^ > 2σ(*F* ^2^)] = 0.056
*wR*(*F* ^2^) = 0.153
*S* = 1.13
7251 reflections
573 parameters
H atoms treated by a mixture of independent and constrained refinement
Δρ_max_ = 0.32 e Å^−3^
Δρ_min_ = −0.31 e Å^−3^

### 

Data collection: *SMART* (Bruker, 2001 ▶); cell refinement: *SAINT-Plus* (Bruker, 2003 ▶); data reduction: *SAINT-Plus*; program(s) used to solve structure: *SHELXS97* (Sheldrick, 2008 ▶); program(s) used to refine structure: *SHELXL97* (Sheldrick, 2008 ▶); molecular graphics: *SHELXTL* (Sheldrick, 2008 ▶); software used to prepare material for publication: *SHELXTL*.

## Supplementary Material

Crystal structure: contains datablocks I, global. DOI: 10.1107/S160053681002341X/rk2209sup1.cif

Structure factors: contains datablocks I. DOI: 10.1107/S160053681002341X/rk2209Isup2.hkl

Additional supplementary materials:  crystallographic information; 3D view; checkCIF report

## Figures and Tables

**Table 1:** Hydrogen-bond geometry (Å, °)

*D*—H⋯*A*	*D*—H	H⋯*A*	*D*⋯*A*	*D*—H⋯*A*
O8*W*—H8*B*⋯O47^i^	0.91 (4)	1.78 (4)	2.658 (3)	162 (4)
O8*W*—H8*A*⋯O1*W*^ii^	0.83 (4)	2.02 (4)	2.809 (3)	159 (4)
O7*W*—H7*A*⋯O12^iii^	0.77 (4)	2.43 (4)	3.068 (3)	141 (4)
O7*W*—H7*B*⋯O35	0.94 (4)	1.73 (4)	2.668 (3)	180 (5)
O6*W*—H6*B*⋯O12^i^	0.84 (4)	2.09 (4)	2.913 (3)	165 (3)
O6*W*—H6*A*⋯O35^i^	0.84 (4)	2.06 (4)	2.843 (3)	156 (3)
O5*W*—H5*B*⋯O47^i^	0.86 (4)	1.96 (4)	2.799 (3)	167 (4)
O5*W*—H5*A*⋯O7*W*^i^	0.76 (4)	2.07 (4)	2.819 (3)	171 (4)
O4*W*—H4*B*⋯O47^iv^	0.86 (4)	1.92 (4)	2.776 (3)	171 (3)
O4*W*—H4*A*⋯O7*W*^v^	0.89 (4)	1.98 (4)	2.854 (3)	164 (3)
O3*W*—H3*B*⋯O8*W*	0.83 (4)	1.98 (4)	2.796 (3)	170 (3)
O3*W*—H3*A*⋯O35	0.82 (4)	2.01 (4)	2.804 (3)	164 (3)
O2*W*—H2*B*⋯O24	0.83 (4)	2.20 (4)	2.932 (3)	148 (3)
O2*W*—H2*A*⋯O47^iv^	0.89 (4)	2.01 (4)	2.870 (3)	164 (3)
O1*W*—H1*B*⋯O8*W*^v^	0.84 (4)	2.08 (4)	2.897 (4)	164 (3)
O1*W*—H1*A*⋯O35^v^	0.91 (4)	1.83 (4)	2.739 (3)	174 (3)
O23—H23⋯O36^vi^	0.84	1.83	2.664 (3)	175
O11—H11⋯O48^vii^	0.84	1.81	2.641 (3)	173

Symmetry codes: (i) 
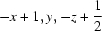
; (ii) 
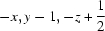
; (iii) 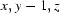; (iv) 
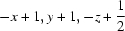
; (v) 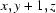; (vi) 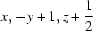; (vii) 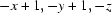.

**Fig. 1.**
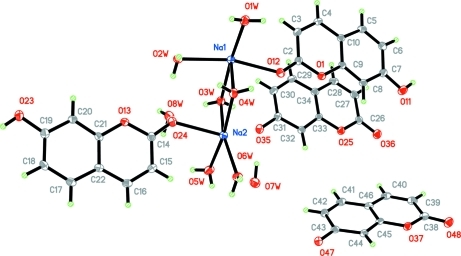
A portion of the infinite chain of the title compound with the atom numbering scheme. Displacement ellipsoids are drawn at 50% probability level. The H atoms are presented as a small spheres of arbitrary radius.

## supplementary crystallographic information

### Comment

It has been suggested that coumarin (1,2-benzopyrone) may be a pro-drug and its major biotransformed product 7-hydroxycoumarin, also known as *umbelliferone*, is an active drug (Egan *et al.*, 1990). The 7-hydroxycoumarin is also a widely distributed natural product which shows *anti*-imflammatory activity (Toyama *et al.*, 2009), and the complexes of coumarin with metal have increased bioactivities such as *anti*-imflammatory activity (Nath *et al.*, 2005). We synthesized the complexe of 7-hydroxycoumarin with sodium for further bioactivities study. This paper is devoted to the crystal structure of the sodium salt of 7-hydroxycoumarin.

The molecular structure of the title compound is shown in Fig. 1. The main moiety of crystal structure is build up of cation chains - (C_18_H_24_Na_2_O_12_)^2+^_n_. The polymeric chain contains two independent crystallographic Na centers. Both Na atoms is six-coordinated by five O atoms of water molecules and one O atom of a ligand. Each 7-hydroxycoumarin ligand links Na centers *via* its O atom of carbonyl group in a chelating manner. Oxygen atoms of two water are bridging atom between two sodium atoms. Na—O bond distances are in the range of 2.288 (2)–2.539 (2)Å. In the chain, two adjacent Na1···Na2 distance of 3.60613 (7)Å. In addition, two 7-hydroxycoumarin anions and two water molecules are hydrogen bonded to the chains.

### Experimental

A stoichiometric amount of NaOH (0.25 mmol) and a quantitative amount of 7-hydroxycoumarin (0.5 mmol) were mixed and then dissolved in ethanol (20 ml). The pH value of the solution was about to 6.5. The solution mixture was stirred continuously for 2 h at room temperature and then filtered. Single crystals were obtained by evaporation after one week.

### Refinement

Hydrogen atoms of the water molecules were located from a difference map and were refined isotropically. The C-bound H-atoms were positioned geometrically and refined using a riding model: C—H = 0.95Å with *U*_iso_(H) = 1.2*U*_eq_(C). The hydroxy O-bound H-atoms were positioned geometrically and refined using a riding model: O—H = 0.84Å with *U*_iso_(H) = 1.5*U*_eq_(O).

### Crystal data

**Table:**

[Na(C_9_H_6_O_3_)(H_2_O)_3_](C_9_H_5_O_3_)·H_2_O	*F*(000) = 1744
*M**_r_* = 418.32	*D*_x_ = 1.491 Mg m^−^^3^
Monoclinic, *P*2/*c*	Mo *K*α radiation, λ = 0.71073 Å
Hall symbol: -P 2yc	Cell parameters from 8701 reflections
*a* = 11.7803 (5) Å	θ = 2.4–27.0°
*b* = 7.6462 (3) Å	µ = 0.14 mm^−^^1^
*c* = 42.3249 (17) Å	*T* = 173 K
β = 102.184 (1)°	Block, yellow
*V* = 3726.5 (3) Å^3^	0.46 × 0.44 × 0.39 mm
*Z* = 8	

### Data collection

**Table:**

Bruker SMART 1000 CCD diffractometer	7251 independent reflections
Radiation source: fine-focus sealed tube	5790 reflections with *I* > 2σ(*I*)
graphite	*R*_int_ = 0.027
φ– and ω–scans	θ_max_ = 26.0°, θ_min_ = 1.0°
Absorption correction: multi-scan (*SADABS*; Sheldrick, 2004)	*h* = −14→10
*T*_min_ = 0.938, *T*_max_ = 0.947	*k* = −9→4
16682 measured reflections	*l* = −43→52

### Refinement

**Table:**

Refinement on *F*^2^	Primary atom site location: structure-invariant direct methods
Least-squares matrix: full	Secondary atom site location: difference Fourier map
*R*[*F*^2^ > 2σ(*F*^2^)] = 0.056	Hydrogen site location: inferred from neighbouring sites
*wR*(*F*^2^) = 0.153	H atoms treated by a mixture of independent and constrained refinement
*S* = 1.13	*w* = 1/[σ^2^(*F*_o_^2^) + (0.0397*P*)^2^ + 6.7016*P*] where *P* = (*F*_o_^2^ + 2*F*_c_^2^)/3
7251 reflections	(Δ/σ)_max_ < 0.001
573 parameters	Δρ_max_ = 0.32 e Å^−^^3^
0 restraints	Δρ_min_ = −0.31 e Å^−^^3^

### Special details

**Table:**

Geometry. All s.u.'s (except the s.u. in the dihedral angle between two l.s. planes) are estimated using the full covariance matrix. The cell s.u.'s are taken into account individually in the estimation of s.u.'s in distances, angles and torsion angles; correlations between s.u.'s in cell parameters are only used when they are defined by crystal symmetry. An approximate (isotropic) treatment of cell s.u.'s is used for estimating s.u.'s involving l.s. planes.
Refinement. Refinement of *F*^2^ against ALL reflections. The weighted *R*-factor *wR* and goodness of fit *S* are based on *F*^2^, conventional *R*-factors *R* are based on *F*, with *F* set to zero for negative *F*^2^. The threshold expression of *F*^2^ > σ(*F*^2^) is used only for calculating *R*-factors(gt) *etc*. and is not relevant to the choice of reflections for refinement. *R*-factors based on *F*^2^ are statistically about twice as large as those based on *F*, and *R*-factors based on ALL data will be even larger.

### Fractional atomic coordinates and isotropic or equivalent isotropic displacement parameters (Å^2^)

**Table:**

	*x*	*y*	*z*	*U*_iso_*/*U*_eq_	
O1	0.23526 (16)	0.8756 (3)	0.13635 (4)	0.0257 (4)	
C2	0.1713 (2)	0.9338 (4)	0.15762 (6)	0.0257 (6)	
C3	0.0611 (2)	1.0154 (4)	0.14468 (7)	0.0281 (6)	
H3	0.0156	1.0592	0.1590	0.034*	
C4	0.0218 (2)	1.0302 (4)	0.11256 (7)	0.0261 (6)	
H4	−0.0505	1.0862	0.1045	0.031*	
C5	0.0514 (2)	0.9665 (4)	0.05654 (7)	0.0291 (6)	
H5	−0.0211	1.0182	0.0470	0.035*	
C6	0.1193 (3)	0.8967 (4)	0.03704 (7)	0.0301 (6)	
H6	0.0940	0.9004	0.0142	0.036*	
C7	0.2261 (2)	0.8199 (4)	0.05101 (7)	0.0279 (6)	
C8	0.2640 (2)	0.8138 (4)	0.08425 (6)	0.0245 (6)	
H8	0.3364	0.7616	0.0937	0.029*	
C9	0.1945 (2)	0.8848 (4)	0.10340 (6)	0.0220 (6)	
C10	0.0873 (2)	0.9629 (4)	0.09044 (6)	0.0235 (6)	
O11	0.29631 (18)	0.7479 (3)	0.03313 (5)	0.0401 (6)	
H11	0.2643	0.7539	0.0134	0.060*	
O12	0.21478 (18)	0.9155 (3)	0.18627 (5)	0.0335 (5)	
O13	0.26587 (16)	0.7216 (3)	0.36781 (4)	0.0262 (4)	
C14	0.3302 (2)	0.6748 (4)	0.34559 (7)	0.0256 (6)	
C15	0.4381 (2)	0.5838 (4)	0.35722 (7)	0.0288 (6)	
H15	0.4838	0.5487	0.3423	0.035*	
C16	0.4752 (2)	0.5479 (4)	0.38881 (7)	0.0270 (6)	
H16	0.5456	0.4854	0.3959	0.032*	
C17	0.4438 (2)	0.5750 (4)	0.44534 (7)	0.0276 (6)	
H17	0.5147	0.5161	0.4538	0.033*	
C18	0.3760 (2)	0.6316 (4)	0.46607 (7)	0.0287 (6)	
H18	0.4001	0.6119	0.4887	0.034*	
C19	0.2712 (2)	0.7186 (4)	0.45381 (7)	0.0278 (6)	
C20	0.2354 (2)	0.7486 (4)	0.42082 (6)	0.0257 (6)	
H20	0.1646	0.8080	0.4124	0.031*	
C21	0.3050 (2)	0.6901 (4)	0.40041 (6)	0.0227 (6)	
C22	0.4097 (2)	0.6028 (3)	0.41194 (7)	0.0236 (6)	
O23	0.20131 (19)	0.7771 (3)	0.47295 (5)	0.0395 (6)	
H23	0.2314	0.7544	0.4923	0.059*	
O24	0.28986 (18)	0.7130 (3)	0.31755 (5)	0.0330 (5)	
O25	0.26302 (16)	0.3348 (3)	0.08489 (4)	0.0278 (4)	
C26	0.2189 (3)	0.3358 (4)	0.05220 (7)	0.0297 (6)	
C27	0.1023 (3)	0.3955 (4)	0.04096 (7)	0.0344 (7)	
H27	0.0697	0.3967	0.0184	0.041*	
C28	0.0378 (3)	0.4502 (4)	0.06194 (7)	0.0331 (7)	
H28	−0.0392	0.4905	0.0540	0.040*	
C29	0.0240 (3)	0.5025 (4)	0.11978 (8)	0.0324 (7)	
H29	−0.0537	0.5432	0.1134	0.039*	
C30	0.0755 (3)	0.4976 (4)	0.15192 (7)	0.0317 (7)	
H30	0.0332	0.5363	0.1674	0.038*	
C31	0.1910 (2)	0.4359 (4)	0.16264 (7)	0.0265 (6)	
C32	0.2516 (2)	0.3821 (4)	0.13896 (7)	0.0255 (6)	
H32	0.3292	0.3408	0.1452	0.031*	
C33	0.1980 (2)	0.3895 (4)	0.10683 (6)	0.0234 (6)	
C34	0.0839 (2)	0.4482 (4)	0.09587 (7)	0.0282 (6)	
O35	0.23967 (17)	0.4289 (3)	0.19366 (5)	0.0304 (5)	
O36	0.28529 (18)	0.2832 (3)	0.03552 (5)	0.0381 (5)	
O37	0.76263 (16)	0.2269 (3)	0.07958 (4)	0.0288 (5)	
C38	0.7208 (3)	0.2060 (4)	0.04703 (7)	0.0304 (7)	
C39	0.6054 (3)	0.1389 (4)	0.03655 (7)	0.0339 (7)	
H39	0.5743	0.1236	0.0141	0.041*	
C40	0.5402 (3)	0.0973 (4)	0.05811 (8)	0.0331 (7)	
H40	0.4638	0.0533	0.0507	0.040*	
C41	0.5241 (3)	0.0783 (4)	0.11626 (8)	0.0334 (7)	
H41	0.4466	0.0363	0.1104	0.040*	
C42	0.5743 (3)	0.0983 (4)	0.14834 (8)	0.0317 (7)	
H42	0.5313	0.0686	0.1642	0.038*	
C43	0.6895 (2)	0.1626 (4)	0.15829 (7)	0.0260 (6)	
C44	0.7499 (2)	0.2065 (4)	0.13408 (7)	0.0260 (6)	
H44	0.8266	0.2521	0.1398	0.031*	
C45	0.6974 (2)	0.1832 (4)	0.10207 (7)	0.0247 (6)	
C46	0.5847 (2)	0.1184 (4)	0.09187 (7)	0.0277 (6)	
O47	0.73790 (17)	0.1803 (3)	0.18917 (5)	0.0320 (5)	
O48	0.78815 (18)	0.2480 (3)	0.02986 (5)	0.0390 (5)	
O1W	0.10600 (19)	1.2312 (3)	0.22568 (6)	0.0340 (5)	
H1A	0.153 (3)	1.290 (5)	0.2146 (8)	0.041*	
H1B	0.119 (3)	1.275 (5)	0.2443 (9)	0.041*	
O2W	0.08624 (18)	0.9228 (3)	0.28844 (5)	0.0296 (5)	
H2A	0.130 (3)	1.013 (5)	0.2967 (8)	0.036*	
H2B	0.122 (3)	0.835 (5)	0.2963 (8)	0.036*	
O3W	0.17663 (18)	0.6416 (3)	0.24113 (5)	0.0288 (5)	
H3A	0.182 (3)	0.584 (5)	0.2253 (8)	0.035*	
H3B	0.148 (3)	0.574 (5)	0.2525 (8)	0.035*	
O4W	0.32795 (19)	0.9807 (3)	0.26292 (5)	0.0306 (5)	
H4A	0.358 (3)	1.057 (5)	0.2509 (8)	0.037*	
H4B	0.315 (3)	1.043 (5)	0.2787 (8)	0.037*	
O5W	0.3969 (2)	0.3917 (3)	0.27914 (6)	0.0382 (6)	
H5A	0.454 (3)	0.344 (5)	0.2824 (9)	0.046*	
H5B	0.358 (3)	0.338 (5)	0.2912 (9)	0.046*	
O6W	0.58882 (18)	0.6911 (3)	0.28677 (5)	0.0293 (5)	
H6A	0.624 (3)	0.602 (5)	0.2951 (8)	0.035*	
H6B	0.636 (3)	0.769 (5)	0.2949 (8)	0.035*	
O7W	0.4008 (2)	0.1863 (4)	0.21454 (6)	0.0451 (6)	
H7A	0.382 (4)	0.104 (6)	0.2044 (10)	0.054*	
H7B	0.344 (3)	0.272 (5)	0.2073 (9)	0.054*	
O8W	0.1042 (2)	0.4173 (4)	0.28519 (6)	0.0444 (6)	
H8A	0.043 (4)	0.370 (5)	0.2869 (9)	0.053*	
H8B	0.159 (3)	0.349 (5)	0.2979 (9)	0.053*	
Na1	0.12359 (9)	0.92812 (14)	0.23211 (3)	0.0257 (3)	
Na2	0.37973 (9)	0.69032 (14)	0.27077 (3)	0.0251 (3)	

### Atomic displacement parameters (Å^2^)

**Table:**

	*U*^11^	*U*^22^	*U*^33^	*U*^12^	*U*^13^	*U*^23^
O1	0.0223 (10)	0.0322 (11)	0.0216 (9)	0.0050 (8)	0.0025 (8)	0.0016 (8)
C2	0.0273 (14)	0.0271 (14)	0.0229 (14)	−0.0006 (12)	0.0062 (11)	−0.0016 (11)
C3	0.0247 (14)	0.0322 (15)	0.0285 (15)	0.0032 (12)	0.0084 (12)	−0.0009 (12)
C4	0.0209 (14)	0.0254 (14)	0.0313 (15)	0.0031 (11)	0.0037 (11)	−0.0020 (12)
C5	0.0232 (14)	0.0316 (16)	0.0294 (15)	0.0035 (12)	−0.0014 (11)	0.0021 (12)
C6	0.0295 (15)	0.0360 (16)	0.0222 (14)	0.0015 (13)	−0.0002 (12)	0.0017 (12)
C7	0.0266 (15)	0.0315 (15)	0.0261 (14)	0.0002 (12)	0.0069 (11)	−0.0013 (12)
C8	0.0184 (13)	0.0273 (14)	0.0270 (14)	0.0026 (11)	0.0026 (11)	0.0012 (12)
C9	0.0214 (13)	0.0227 (13)	0.0204 (13)	−0.0019 (11)	0.0014 (10)	0.0010 (11)
C10	0.0196 (13)	0.0243 (14)	0.0257 (14)	0.0004 (11)	0.0032 (11)	0.0013 (11)
O11	0.0342 (12)	0.0614 (16)	0.0245 (10)	0.0128 (11)	0.0057 (9)	−0.0046 (11)
O12	0.0357 (12)	0.0416 (13)	0.0224 (10)	0.0074 (10)	0.0042 (9)	0.0032 (9)
O13	0.0241 (10)	0.0314 (11)	0.0225 (10)	0.0052 (8)	0.0034 (8)	0.0023 (8)
C14	0.0275 (14)	0.0242 (14)	0.0258 (14)	−0.0016 (12)	0.0071 (11)	0.0004 (11)
C15	0.0259 (15)	0.0279 (15)	0.0339 (15)	0.0017 (12)	0.0089 (12)	−0.0019 (12)
C16	0.0196 (13)	0.0249 (14)	0.0351 (15)	−0.0011 (11)	0.0025 (11)	0.0021 (12)
C17	0.0222 (14)	0.0282 (15)	0.0297 (15)	−0.0002 (12)	−0.0011 (11)	0.0029 (12)
C18	0.0290 (15)	0.0325 (16)	0.0222 (14)	−0.0032 (13)	−0.0002 (11)	0.0055 (12)
C19	0.0269 (14)	0.0307 (15)	0.0258 (14)	−0.0034 (12)	0.0054 (11)	−0.0004 (12)
C20	0.0190 (13)	0.0304 (15)	0.0263 (14)	0.0009 (12)	0.0017 (11)	0.0009 (12)
C21	0.0209 (13)	0.0238 (13)	0.0216 (13)	−0.0037 (11)	0.0008 (10)	0.0029 (11)
C22	0.0208 (13)	0.0188 (13)	0.0299 (14)	−0.0028 (11)	0.0026 (11)	0.0020 (11)
O23	0.0358 (12)	0.0591 (15)	0.0242 (10)	0.0091 (11)	0.0078 (9)	0.0017 (11)
O24	0.0351 (11)	0.0403 (12)	0.0234 (10)	0.0050 (10)	0.0059 (9)	0.0011 (9)
O25	0.0218 (10)	0.0370 (11)	0.0248 (10)	0.0010 (9)	0.0051 (8)	0.0004 (9)
C26	0.0284 (15)	0.0350 (16)	0.0248 (14)	−0.0039 (13)	0.0036 (12)	0.0028 (12)
C27	0.0323 (16)	0.0403 (18)	0.0269 (15)	0.0021 (14)	−0.0027 (12)	0.0065 (13)
C28	0.0243 (15)	0.0323 (16)	0.0392 (17)	0.0038 (13)	−0.0009 (13)	0.0041 (14)
C29	0.0252 (15)	0.0283 (15)	0.0449 (18)	0.0045 (12)	0.0105 (13)	0.0026 (13)
C30	0.0308 (16)	0.0290 (15)	0.0387 (17)	0.0020 (13)	0.0149 (13)	−0.0026 (13)
C31	0.0289 (15)	0.0225 (14)	0.0291 (15)	−0.0051 (12)	0.0086 (12)	−0.0017 (12)
C32	0.0186 (13)	0.0284 (15)	0.0296 (14)	0.0000 (11)	0.0051 (11)	0.0009 (12)
C33	0.0224 (14)	0.0218 (13)	0.0270 (14)	−0.0011 (11)	0.0073 (11)	0.0006 (11)
C34	0.0233 (14)	0.0258 (14)	0.0353 (16)	0.0001 (12)	0.0055 (12)	0.0033 (12)
O35	0.0331 (11)	0.0320 (11)	0.0273 (10)	−0.0018 (9)	0.0093 (9)	−0.0019 (9)
O36	0.0334 (12)	0.0544 (15)	0.0267 (11)	0.0030 (11)	0.0067 (9)	0.0000 (10)
O37	0.0208 (10)	0.0409 (12)	0.0239 (10)	0.0017 (9)	0.0027 (8)	−0.0034 (9)
C38	0.0297 (15)	0.0353 (16)	0.0241 (14)	0.0079 (13)	0.0010 (12)	−0.0061 (12)
C39	0.0311 (16)	0.0363 (17)	0.0293 (15)	0.0037 (14)	−0.0047 (12)	−0.0078 (13)
C40	0.0237 (15)	0.0307 (16)	0.0415 (17)	0.0017 (13)	−0.0009 (13)	−0.0039 (13)
C41	0.0210 (14)	0.0297 (16)	0.0485 (19)	−0.0015 (12)	0.0048 (13)	−0.0023 (14)
C42	0.0290 (15)	0.0261 (15)	0.0420 (17)	0.0008 (12)	0.0120 (13)	0.0063 (13)
C43	0.0262 (14)	0.0207 (14)	0.0311 (15)	0.0042 (11)	0.0065 (12)	0.0002 (11)
C44	0.0193 (13)	0.0292 (15)	0.0289 (14)	0.0004 (11)	0.0036 (11)	−0.0005 (12)
C45	0.0207 (13)	0.0259 (14)	0.0277 (14)	0.0049 (11)	0.0056 (11)	−0.0023 (12)
C46	0.0218 (14)	0.0237 (14)	0.0355 (16)	0.0022 (11)	0.0015 (12)	−0.0040 (12)
O47	0.0313 (11)	0.0371 (12)	0.0286 (11)	0.0023 (9)	0.0086 (9)	0.0042 (9)
O48	0.0322 (12)	0.0584 (15)	0.0269 (11)	0.0011 (11)	0.0071 (9)	−0.0055 (10)
O1W	0.0356 (12)	0.0305 (12)	0.0401 (12)	−0.0022 (10)	0.0175 (10)	−0.0003 (10)
O2W	0.0251 (11)	0.0322 (12)	0.0304 (11)	0.0033 (9)	0.0030 (8)	0.0006 (9)
O3W	0.0342 (12)	0.0238 (11)	0.0300 (11)	−0.0007 (9)	0.0101 (9)	0.0003 (9)
O4W	0.0380 (12)	0.0269 (11)	0.0298 (11)	−0.0024 (9)	0.0139 (9)	0.0000 (9)
O5W	0.0362 (13)	0.0244 (11)	0.0600 (15)	0.0047 (10)	0.0233 (12)	0.0088 (10)
O6W	0.0242 (11)	0.0293 (11)	0.0325 (11)	−0.0018 (9)	0.0017 (9)	−0.0006 (9)
O7W	0.0342 (13)	0.0483 (15)	0.0520 (16)	−0.0033 (12)	0.0073 (11)	0.0151 (12)
O8W	0.0278 (12)	0.0590 (16)	0.0465 (14)	−0.0053 (12)	0.0082 (10)	0.0183 (12)
Na1	0.0269 (6)	0.0251 (6)	0.0259 (6)	0.0026 (5)	0.0074 (4)	0.0019 (4)
Na2	0.0245 (6)	0.0259 (6)	0.0253 (5)	0.0003 (5)	0.0059 (4)	0.0010 (4)

### Geometric parameters (Å, °)

**Table:**

O1—C2	1.365 (3)	C30—H30	0.9500
O1—C9	1.378 (3)	C31—O35	1.318 (3)
C2—O12	1.221 (3)	C31—C32	1.410 (4)
C2—C3	1.440 (4)	C32—C33	1.375 (4)
C3—C4	1.345 (4)	C32—H32	0.9500
C3—H3	0.9500	C33—C34	1.400 (4)
C4—C10	1.429 (4)	O37—C38	1.371 (3)
C4—H4	0.9500	O37—C45	1.385 (3)
C5—C6	1.373 (4)	C38—O48	1.226 (4)
C5—C10	1.408 (4)	C38—C39	1.433 (4)
C5—H5	0.9500	C39—C40	1.348 (4)
C6—C7	1.401 (4)	C39—H39	0.9500
C6—H6	0.9500	C40—C46	1.423 (4)
C7—O11	1.351 (3)	C40—H40	0.9500
C7—C8	1.384 (4)	C41—C42	1.370 (4)
C8—C9	1.379 (4)	C41—C46	1.407 (4)
C8—H8	0.9500	C41—H41	0.9500
C9—C10	1.400 (4)	C42—C43	1.421 (4)
O11—H11	0.8400	C42—H42	0.9500
O12—Na1	2.410 (2)	C43—O47	1.318 (3)
O13—C14	1.374 (3)	C43—C44	1.407 (4)
O13—C21	1.380 (3)	C44—C45	1.377 (4)
C14—O24	1.217 (3)	C44—H44	0.9500
C14—C15	1.442 (4)	C45—C46	1.397 (4)
C15—C16	1.344 (4)	O1W—Na1	2.338 (2)
C15—H15	0.9500	O1W—H1A	0.91 (4)
C16—C22	1.431 (4)	O1W—H1B	0.84 (4)
C16—H16	0.9500	O2W—Na1^i^	2.442 (2)
C17—C18	1.375 (4)	O2W—Na1	2.514 (2)
C17—C22	1.402 (4)	O2W—H2A	0.89 (4)
C17—H17	0.9500	O2W—H2B	0.83 (4)
C18—C19	1.402 (4)	O3W—Na1	2.288 (2)
C18—H18	0.9500	O3W—Na2	2.484 (2)
C19—O23	1.347 (3)	O3W—H3A	0.82 (4)
C19—C20	1.390 (4)	O3W—H3B	0.83 (4)
C20—C21	1.385 (4)	O4W—Na2	2.308 (2)
C20—H20	0.9500	O4W—Na1	2.516 (2)
C21—C22	1.397 (4)	O4W—H4A	0.89 (4)
O23—H23	0.8400	O4W—H4B	0.86 (4)
O24—Na2	2.440 (2)	O5W—Na2	2.313 (2)
O25—C26	1.372 (3)	O5W—H5A	0.76 (4)
O25—C33	1.387 (3)	O5W—H5B	0.86 (4)
C26—O36	1.226 (4)	O6W—Na2	2.413 (2)
C26—C27	1.431 (4)	O6W—Na2^ii^	2.539 (2)
C27—C28	1.351 (4)	O6W—H6A	0.84 (4)
C27—H27	0.9500	O6W—H6B	0.84 (4)
C28—C34	1.424 (4)	O7W—H7A	0.77 (4)
C28—H28	0.9500	O7W—H7B	0.94 (4)
C29—C30	1.368 (4)	O8W—H8A	0.83 (4)
C29—C34	1.413 (4)	O8W—H8B	0.91 (4)
C29—H29	0.9500	Na1—O2W^i^	2.442 (2)
C30—C31	1.421 (4)	Na2—O6W^ii^	2.539 (2)
			
C2—O1—C9	121.9 (2)	C41—C42—H42	119.4
O12—C2—O1	116.3 (2)	C43—C42—H42	119.4
O12—C2—C3	125.7 (3)	O47—C43—C44	121.2 (3)
O1—C2—C3	118.0 (2)	O47—C43—C42	121.0 (3)
C4—C3—C2	120.7 (3)	C44—C43—C42	117.8 (3)
C4—C3—H3	119.6	C45—C44—C43	119.6 (3)
C2—C3—H3	119.6	C45—C44—H44	120.2
C3—C4—C10	120.9 (3)	C43—C44—H44	120.2
C3—C4—H4	119.5	C44—C45—O37	116.4 (2)
C10—C4—H4	119.5	C44—C45—C46	123.4 (3)
C6—C5—C10	121.1 (3)	O37—C45—C46	120.2 (2)
C6—C5—H5	119.4	C45—C46—C41	116.5 (3)
C10—C5—H5	119.4	C45—C46—C40	118.4 (3)
C5—C6—C7	119.6 (3)	C41—C46—C40	125.1 (3)
C5—C6—H6	120.2	Na1—O1W—H1A	120 (2)
C7—C6—H6	120.2	Na1—O1W—H1B	107 (3)
O11—C7—C8	116.8 (3)	H1A—O1W—H1B	106 (3)
O11—C7—C6	122.4 (3)	Na1^i^—O2W—Na1	91.63 (8)
C8—C7—C6	120.7 (3)	Na1^i^—O2W—H2A	126 (2)
C9—C8—C7	118.7 (3)	Na1—O2W—H2A	99 (2)
C9—C8—H8	120.7	Na1^i^—O2W—H2B	124 (2)
C7—C8—H8	120.7	Na1—O2W—H2B	102 (2)
O1—C9—C8	116.9 (2)	H2A—O2W—H2B	105 (3)
O1—C9—C10	120.7 (2)	Na1—O3W—Na2	98.08 (9)
C8—C9—C10	122.4 (2)	Na1—O3W—H3A	116 (2)
C9—C10—C5	117.4 (2)	Na2—O3W—H3A	105 (2)
C9—C10—C4	117.7 (2)	Na1—O3W—H3B	124 (2)
C5—C10—C4	125.0 (3)	Na2—O3W—H3B	106 (2)
C7—O11—H11	109.5	H3A—O3W—H3B	105 (3)
C2—O12—Na1	129.07 (19)	Na2—O4W—Na1	96.65 (8)
C14—O13—C21	121.5 (2)	Na2—O4W—H4A	126 (2)
O24—C14—O13	116.5 (2)	Na1—O4W—H4A	105 (2)
O24—C14—C15	125.7 (3)	Na2—O4W—H4B	120 (2)
O13—C14—C15	117.8 (2)	Na1—O4W—H4B	101 (2)
C16—C15—C14	121.1 (3)	H4A—O4W—H4B	103 (3)
C16—C15—H15	119.5	Na2—O5W—H5A	124 (3)
C14—C15—H15	119.5	Na2—O5W—H5B	122 (3)
C15—C16—C22	120.8 (3)	H5A—O5W—H5B	103 (4)
C15—C16—H16	119.6	Na2—O6W—Na2^ii^	94.47 (8)
C22—C16—H16	119.6	Na2—O6W—H6A	120 (2)
C18—C17—C22	121.0 (3)	Na2^ii^—O6W—H6A	104 (2)
C18—C17—H17	119.5	Na2—O6W—H6B	132 (2)
C22—C17—H17	119.5	Na2^ii^—O6W—H6B	100 (2)
C17—C18—C19	119.9 (3)	H6A—O6W—H6B	101 (3)
C17—C18—H18	120.1	H7A—O7W—H7B	107 (4)
C19—C18—H18	120.1	H8A—O8W—H8B	102 (4)
O23—C19—C20	117.0 (3)	O3W—Na1—O1W	169.46 (10)
O23—C19—C18	122.5 (3)	O3W—Na1—O12	86.77 (8)
C20—C19—C18	120.5 (3)	O1W—Na1—O12	89.40 (8)
C21—C20—C19	118.7 (3)	O3W—Na1—O2W^i^	105.29 (9)
C21—C20—H20	120.7	O1W—Na1—O2W^i^	85.23 (9)
C19—C20—H20	120.7	O12—Na1—O2W^i^	107.59 (8)
O13—C21—C20	116.8 (2)	O3W—Na1—O2W	85.56 (8)
O13—C21—C22	121.1 (2)	O1W—Na1—O2W	95.60 (9)
C20—C21—C22	122.1 (2)	O12—Na1—O2W	163.68 (8)
C21—C22—C17	117.9 (3)	O2W^i^—Na1—O2W	88.34 (8)
C21—C22—C16	117.7 (2)	O3W—Na1—O4W	82.46 (8)
C17—C22—C16	124.4 (3)	O1W—Na1—O4W	87.34 (8)
C19—O23—H23	109.5	O12—Na1—O4W	83.30 (8)
C14—O24—Na2	128.84 (19)	O2W^i^—Na1—O4W	166.71 (9)
C26—O25—C33	121.9 (2)	O2W—Na1—O4W	81.43 (8)
O36—C26—O25	115.3 (3)	O3W—Na1—Na1^i^	98.53 (6)
O36—C26—C27	126.7 (3)	O1W—Na1—Na1^i^	89.38 (6)
O25—C26—C27	118.0 (3)	O12—Na1—Na1^i^	152.55 (8)
C28—C27—C26	120.9 (3)	O2W^i^—Na1—Na1^i^	45.00 (5)
C28—C27—H27	119.5	O2W—Na1—Na1^i^	43.37 (5)
C26—C27—H27	119.5	O4W—Na1—Na1^i^	124.02 (7)
C27—C28—C34	120.8 (3)	O3W—Na1—Na2	43.00 (6)
C27—C28—H28	119.6	O1W—Na1—Na2	126.72 (7)
C34—C28—H28	119.6	O12—Na1—Na2	82.68 (6)
C30—C29—C34	121.3 (3)	O2W^i^—Na1—Na2	147.20 (7)
C30—C29—H29	119.3	O2W—Na1—Na2	81.88 (6)
C34—C29—H29	119.3	O4W—Na1—Na2	39.47 (5)
C29—C30—C31	121.4 (3)	Na1^i^—Na1—Na2	119.28 (4)
C29—C30—H30	119.3	O4W—Na2—O5W	169.64 (10)
C31—C30—H30	119.3	O4W—Na2—O6W	105.13 (9)
O35—C31—C32	121.1 (3)	O5W—Na2—O6W	84.73 (9)
O35—C31—C30	121.2 (3)	O4W—Na2—O24	84.36 (8)
C32—C31—C30	117.7 (3)	O5W—Na2—O24	89.13 (9)
C33—C32—C31	119.7 (3)	O6W—Na2—O24	111.35 (8)
C33—C32—H32	120.2	O4W—Na2—O3W	82.79 (8)
C31—C32—H32	120.2	O5W—Na2—O3W	88.46 (9)
C32—C33—O25	116.5 (2)	O6W—Na2—O3W	163.64 (9)
C32—C33—C34	123.3 (3)	O24—Na2—O3W	83.33 (8)
O25—C33—C34	120.1 (2)	O4W—Na2—O6W^ii^	86.98 (8)
C33—C34—C29	116.6 (3)	O5W—Na2—O6W^ii^	97.09 (9)
C33—C34—C28	118.2 (3)	O6W—Na2—O6W^ii^	85.53 (8)
C29—C34—C28	125.2 (3)	O24—Na2—O6W^ii^	162.54 (8)
C38—O37—C45	121.9 (2)	O3W—Na2—O6W^ii^	80.56 (7)
O48—C38—O37	115.1 (3)	O4W—Na2—Na1	43.88 (6)
O48—C38—C39	126.9 (3)	O5W—Na2—Na1	127.24 (8)
O37—C38—C39	117.9 (3)	O6W—Na2—Na1	146.40 (7)
C40—C39—C38	120.9 (3)	O24—Na2—Na1	82.39 (6)
C40—C39—H39	119.6	O3W—Na2—Na1	38.92 (5)
C38—C39—H39	119.6	O6W^ii^—Na2—Na1	80.86 (6)
C39—C40—C46	120.7 (3)	O4W—Na2—Na2^ii^	98.02 (6)
C39—C40—H40	119.6	O5W—Na2—Na2^ii^	91.26 (6)
C46—C40—H40	119.6	O6W—Na2—Na2^ii^	44.12 (5)
C42—C41—C46	121.5 (3)	O24—Na2—Na2^ii^	155.23 (7)
C42—C41—H41	119.2	O3W—Na2—Na2^ii^	121.44 (7)
C46—C41—H41	119.2	O6W^ii^—Na2—Na2^ii^	41.41 (5)
C41—C42—C43	121.1 (3)	Na1—Na2—Na2^ii^	116.32 (4)
			
C9—O1—C2—O12	178.3 (2)	Na2—O3W—Na1—O2W	−82.85 (8)
C9—O1—C2—C3	−3.5 (4)	Na2—O3W—Na1—O4W	−0.92 (8)
O12—C2—C3—C4	179.3 (3)	Na2—O3W—Na1—Na1^i^	−124.35 (7)
O1—C2—C3—C4	1.3 (4)	C2—O12—Na1—O3W	117.1 (3)
C2—C3—C4—C10	1.1 (4)	C2—O12—Na1—O1W	−72.7 (3)
C10—C5—C6—C7	0.2 (5)	C2—O12—Na1—O2W^i^	12.1 (3)
C5—C6—C7—O11	179.5 (3)	C2—O12—Na1—O2W	179.2 (3)
C5—C6—C7—C8	0.0 (5)	C2—O12—Na1—O4W	−160.1 (3)
O11—C7—C8—C9	−179.6 (3)	C2—O12—Na1—Na1^i^	14.8 (3)
C6—C7—C8—C9	0.0 (4)	C2—O12—Na1—Na2	160.1 (3)
C2—O1—C9—C8	−176.7 (2)	Na1^i^—O2W—Na1—O3W	−107.39 (9)
C2—O1—C9—C10	3.2 (4)	Na1^i^—O2W—Na1—O1W	83.13 (9)
C7—C8—C9—O1	179.9 (2)	Na1^i^—O2W—Na1—O12	−169.6 (3)
C7—C8—C9—C10	−0.1 (4)	Na1^i^—O2W—Na1—O2W^i^	−1.91 (12)
O1—C9—C10—C5	−179.7 (2)	Na1^i^—O2W—Na1—O4W	169.58 (8)
C8—C9—C10—C5	0.2 (4)	Na1^i^—O2W—Na1—Na2	−150.51 (7)
O1—C9—C10—C4	−0.7 (4)	Na2—O4W—Na1—O3W	0.99 (9)
C8—C9—C10—C4	179.2 (3)	Na2—O4W—Na1—O1W	−176.33 (9)
C6—C5—C10—C9	−0.2 (4)	Na2—O4W—Na1—O12	−86.62 (9)
C6—C5—C10—C4	−179.1 (3)	Na2—O4W—Na1—O2W^i^	127.7 (4)
C3—C4—C10—C9	−1.4 (4)	Na2—O4W—Na1—O2W	87.60 (9)
C3—C4—C10—C5	177.5 (3)	Na2—O4W—Na1—Na1^i^	96.23 (7)
O1—C2—O12—Na1	−163.33 (18)	Na1—O4W—Na2—O5W	−33.5 (6)
C3—C2—O12—Na1	18.6 (4)	Na1—O4W—Na2—O6W	164.49 (8)
C21—O13—C14—O24	−177.8 (2)	Na1—O4W—Na2—O24	−84.88 (8)
C21—O13—C14—C15	2.8 (4)	Na1—O4W—Na2—O3W	−0.91 (8)
O24—C14—C15—C16	179.9 (3)	Na1—O4W—Na2—O6W^ii^	79.95 (8)
O13—C14—C15—C16	−0.8 (4)	Na1—O4W—Na2—Na2^ii^	119.96 (7)
C14—C15—C16—C22	−1.5 (4)	Na2^ii^—O6W—Na2—O4W	−85.92 (9)
C22—C17—C18—C19	0.0 (4)	Na2^ii^—O6W—Na2—O5W	97.29 (9)
C17—C18—C19—O23	−180.0 (3)	Na2^ii^—O6W—Na2—O24	−175.72 (7)
C17—C18—C19—C20	−0.2 (4)	Na2^ii^—O6W—Na2—O3W	31.5 (4)
O23—C19—C20—C21	−179.9 (3)	Na2^ii^—O6W—Na2—O6W^ii^	−0.27 (12)
C18—C19—C20—C21	0.3 (4)	Na2^ii^—O6W—Na2—Na1	−66.35 (14)
C14—O13—C21—C20	177.6 (2)	C14—O24—Na2—O4W	−127.3 (3)
C14—O13—C21—C22	−2.6 (4)	C14—O24—Na2—O5W	60.7 (3)
C19—C20—C21—O13	179.6 (2)	C14—O24—Na2—O6W	−23.3 (3)
C19—C20—C21—C22	−0.2 (4)	C14—O24—Na2—O3W	149.3 (3)
O13—C21—C22—C17	−179.8 (2)	C14—O24—Na2—O6W^ii^	172.1 (3)
C20—C21—C22—C17	0.0 (4)	C14—O24—Na2—Na1	−171.5 (3)
O13—C21—C22—C16	0.3 (4)	C14—O24—Na2—Na2^ii^	−30.4 (3)
C20—C21—C22—C16	−179.9 (3)	Na1—O3W—Na2—O4W	1.01 (9)
C18—C17—C22—C21	0.1 (4)	Na1—O3W—Na2—O5W	175.44 (10)
C18—C17—C22—C16	−180.0 (3)	Na1—O3W—Na2—O6W	−119.2 (3)
C15—C16—C22—C21	1.7 (4)	Na1—O3W—Na2—O24	86.13 (9)
C15—C16—C22—C17	−178.2 (3)	Na1—O3W—Na2—O6W^ii^	−87.11 (9)
O13—C14—O24—Na2	171.97 (17)	Na1—O3W—Na2—Na2^ii^	−94.02 (7)
C15—C14—O24—Na2	−8.7 (4)	O3W—Na1—Na2—O4W	−178.56 (12)
C33—O25—C26—O36	−179.6 (3)	O1W—Na1—Na2—O4W	4.58 (11)
C33—O25—C26—C27	0.0 (4)	O12—Na1—Na2—O4W	88.33 (10)
O36—C26—C27—C28	179.9 (3)	O2W^i^—Na1—Na2—O4W	−160.37 (15)
O25—C26—C27—C28	0.4 (5)	O2W—Na1—Na2—O4W	−86.36 (10)
C26—C27—C28—C34	−0.7 (5)	Na1^i^—Na1—Na2—O4W	−109.16 (9)
C34—C29—C30—C31	−0.7 (5)	O3W—Na1—Na2—O5W	−5.73 (12)
C29—C30—C31—O35	−178.9 (3)	O1W—Na1—Na2—O5W	177.41 (11)
C29—C30—C31—C32	1.0 (4)	O12—Na1—Na2—O5W	−98.84 (11)
O35—C31—C32—C33	179.4 (3)	O2W^i^—Na1—Na2—O5W	12.46 (16)
C30—C31—C32—C33	−0.5 (4)	O2W—Na1—Na2—O5W	86.47 (11)
C31—C32—C33—O25	179.9 (2)	O4W—Na1—Na2—O5W	172.83 (13)
C31—C32—C33—C34	−0.2 (4)	Na1^i^—Na1—Na2—O5W	63.67 (10)
C26—O25—C33—C32	179.8 (3)	O3W—Na1—Na2—O6W	153.64 (15)
C26—O25—C33—C34	−0.1 (4)	O1W—Na1—Na2—O6W	−23.23 (16)
C32—C33—C34—C29	0.4 (4)	O12—Na1—Na2—O6W	60.52 (13)
O25—C33—C34—C29	−179.6 (3)	O2W^i^—Na1—Na2—O6W	171.83 (15)
C32—C33—C34—C28	179.9 (3)	O2W—Na1—Na2—O6W	−114.17 (13)
O25—C33—C34—C28	−0.2 (4)	O4W—Na1—Na2—O6W	−27.80 (14)
C30—C29—C34—C33	0.0 (4)	Na1^i^—Na1—Na2—O6W	−136.96 (11)
C30—C29—C34—C28	−179.4 (3)	O3W—Na1—Na2—O24	−88.79 (10)
C27—C28—C34—C33	0.6 (4)	O1W—Na1—Na2—O24	94.34 (10)
C27—C28—C34—C29	180.0 (3)	O12—Na1—Na2—O24	178.09 (8)
C45—O37—C38—O48	−179.2 (3)	O2W^i^—Na1—Na2—O24	−70.60 (13)
C45—O37—C38—C39	0.9 (4)	O2W—Na1—Na2—O24	3.40 (8)
O48—C38—C39—C40	179.9 (3)	O4W—Na1—Na2—O24	89.77 (10)
O37—C38—C39—C40	−0.2 (4)	Na1^i^—Na1—Na2—O24	−19.40 (7)
C38—C39—C40—C46	−0.2 (5)	O1W—Na1—Na2—O3W	−176.86 (13)
C46—C41—C42—C43	0.8 (5)	O12—Na1—Na2—O3W	−93.11 (10)
C41—C42—C43—O47	−179.4 (3)	O2W^i^—Na1—Na2—O3W	18.19 (14)
C41—C42—C43—C44	0.5 (4)	O2W—Na1—Na2—O3W	92.20 (10)
O47—C43—C44—C45	178.8 (3)	O4W—Na1—Na2—O3W	178.56 (12)
C42—C43—C44—C45	−1.2 (4)	Na1^i^—Na1—Na2—O3W	69.40 (9)
C43—C44—C45—O37	−179.1 (2)	O3W—Na1—Na2—O6W^ii^	86.26 (10)
C43—C44—C45—C46	0.6 (4)	O1W—Na1—Na2—O6W^ii^	−90.60 (10)
C38—O37—C45—C44	178.6 (3)	O12—Na1—Na2—O6W^ii^	−6.85 (8)
C38—O37—C45—C46	−1.1 (4)	O2W^i^—Na1—Na2—O6W^ii^	104.45 (13)
C44—C45—C46—C41	0.7 (4)	O2W—Na1—Na2—O6W^ii^	178.46 (8)
O37—C45—C46—C41	−179.6 (3)	O4W—Na1—Na2—O6W^ii^	−95.18 (10)
C44—C45—C46—C40	−179.0 (3)	Na1^i^—Na1—Na2—O6W^ii^	155.66 (6)
O37—C45—C46—C40	0.7 (4)	O3W—Na1—Na2—Na2^ii^	108.28 (9)
C42—C41—C46—C45	−1.4 (4)	O1W—Na1—Na2—Na2^ii^	−68.58 (9)
C42—C41—C46—C40	178.3 (3)	O12—Na1—Na2—Na2^ii^	15.17 (7)
C39—C40—C46—C45	−0.1 (4)	O2W^i^—Na1—Na2—Na2^ii^	126.47 (12)
C39—C40—C46—C41	−179.7 (3)	O2W—Na1—Na2—Na2^ii^	−159.52 (6)
Na2—O3W—Na1—O1W	13.9 (6)	O4W—Na1—Na2—Na2^ii^	−73.16 (8)
Na2—O3W—Na1—O12	82.74 (9)	Na1^i^—Na1—Na2—Na2^ii^	177.68 (3)
Na2—O3W—Na1—O2W^i^	−169.90 (8)		

Symmetry codes: (i) −*x*, *y*, −*z*+1/2; (ii) −*x*+1, *y*, −*z*+1/2.

### Hydrogen-bond geometry (Å, °)

**Table:**

*D*—H···*A*	*D*—H	H···*A*	*D*···*A*	*D*—H···*A*
O8W—H8B···O47^ii^	0.91 (4)	1.78 (4)	2.658 (3)	162 (4)
O8W—H8A···O1W^iii^	0.83 (4)	2.02 (4)	2.809 (3)	159 (4)
O7W—H7A···O12^iv^	0.77 (4)	2.43 (4)	3.068 (3)	141 (4)
O7W—H7B···O35	0.94 (4)	1.73 (4)	2.668 (3)	180 (5)
O6W—H6B···O12^ii^	0.84 (4)	2.09 (4)	2.913 (3)	165 (3)
O6W—H6A···O35^ii^	0.84 (4)	2.06 (4)	2.843 (3)	156 (3)
O5W—H5B···O47^ii^	0.86 (4)	1.96 (4)	2.799 (3)	167 (4)
O5W—H5A···O7W^ii^	0.76 (4)	2.07 (4)	2.819 (3)	171 (4)
O4W—H4B···O47^v^	0.86 (4)	1.92 (4)	2.776 (3)	171 (3)
O4W—H4A···O7W^vi^	0.89 (4)	1.98 (4)	2.854 (3)	164 (3)
O3W—H3B···O8W	0.83 (4)	1.98 (4)	2.796 (3)	170 (3)
O3W—H3A···O35	0.82 (4)	2.01 (4)	2.804 (3)	164 (3)
O2W—H2B···O24	0.83 (4)	2.20 (4)	2.932 (3)	148 (3)
O2W—H2A···O47^v^	0.89 (4)	2.01 (4)	2.870 (3)	164 (3)
O1W—H1B···O8W^vi^	0.84 (4)	2.08 (4)	2.897 (4)	164 (3)
O1W—H1A···O35^vi^	0.91 (4)	1.83 (4)	2.739 (3)	174 (3)
O23—H23···O36^vii^	0.84	1.83	2.664 (3)	175
O11—H11···O48^viii^	0.84	1.81	2.641 (3)	173

Symmetry codes: (ii) −*x*+1, *y*, −*z*+1/2; (iii) −*x*, *y*−1, −*z*+1/2; (iv) *x*, *y*−1, *z*; (v) −*x*+1, *y*+1, −*z*+1/2; (vi) *x*, *y*+1, *z*; (vii) *x*, −*y*+1, *z*+1/2; (viii) −*x*+1, −*y*+1, −*z*.
